# An Imprinted Cross-Linked Enzyme Aggregate (iCLEA) of Sucrose Phosphorylase: Combining Improved Stability with Altered Specificity

**DOI:** 10.3390/ijms130911333

**Published:** 2012-09-11

**Authors:** Karel De Winter, Wim Soetaert, Tom Desmet

**Affiliations:** Centre of Expertise for Industrial Biotechnology and Biocatalysis, Department of Biochemical and Microbial Technology, Faculty of Biosciences Engineering, Ghent University, Coupure Links 653, Ghent B-9000, Belgium; E-Mails: karel.dewinter@ugent.be (K.D.W.); wim.soetaert@ugent.be (W.S.)

**Keywords:** sucrose phosphorylase, immobilization, imprinting, CLEA, glucosyl glycerol

## Abstract

The industrial use of sucrose phosphorylase (SP), an interesting biocatalyst for the selective transfer of α-glucosyl residues to various acceptor molecules, has been hampered by a lack of long-term stability and low activity towards alternative substrates. We have recently shown that the stability of the SP from *Bifidobacterium adolescentis* can be significantly improved by the formation of a cross-linked enzyme aggregate (CLEA). In this work, it is shown that the transglucosylation activity of such a CLEA can also be improved by molecular imprinting with a suitable substrate. To obtain proof of concept, SP was imprinted with α-glucosyl glycerol and subsequently cross-linked with glutaraldehyde. As a consequence, the enzyme’s specific activity towards glycerol as acceptor substrate was increased two-fold while simultaneously providing an exceptional stability at 60 °C. This procedure can be performed in an aqueous environment and gives rise to a new enzyme formulation called iCLEA.

## 1. Introduction

Sucrose phosphorylase (SP) catalyzes the reversible phosphorolysis of sucrose into α-d-glucose 1-phosphate (αG1P) and d-fructose. Although it is formally classified as a glycosyl transferase (EC 2.4.1.7), the enzyme belongs to glycoside hydrolase family 13 [1] and follows the typical double displacement mechanism of retaining glycosidases [2]. The crystal structure of the enzyme from *Bifidobacterium adolescentis* has been determined and consists of a (β/α)_8_ barrel containing Asp192 (nucleophile) and Glu232 (acid/base) as catalytic residues [3]. Thanks to its broad acceptor specificity, SP can be employed for the transfer of glucose to a wide variety of carbohydrates as well as non-carbohydrate molecules [4]. For example, an exceptionally efficient process for the production of α-glucosyl glycerol (αGG) has been developed with SP [5,6]. The product is a moisturizing agent for cosmetics and is commercially available under the tradename Glycoin^®^.

Unfortunately, the use of SP as industrial biocatalyst has been limited by a lack of long-term stability and low activity on alternative substrates [7,8]. Very recently, however, significant improvements in the thermostability of the SP from *B. adolescentis* have been realized by immobilization on Sepabeads [9], formation of a cross-linked enzyme aggregate (CLEA) [10,11] and introduction of specific mutations [12]. In contrast, an enzyme with a modified specificity has not yet been reported, although studies aiming to alter the acceptor specificity of SP by protein engineering have revealed insight into the glycosylation mechanism [13] and succeeded to suppress the hydrolytic activity without affecting the ability of phenolic compounds to act as acceptor [14].

Molecular imprinting is an attractive alternative to mutagenesis for the manipulation of enzymatic properties [15]. Indeed, the three-dimensional structure of a protein can be modified by noncovalent interactions with an imprinting molecule in mild denaturing conditions [16]. Unfortunately, imprinted enzymes are only able to maintain their new conformation when transferred to a non-aqueous environment or when immobilized by cross-linking [17,18]. For the latter strategy, a radical polymerization procedure has been reported that requires transfer to organic solvents and derivatization of the enzyme [19–21].

In this work, we describe the molecular imprinting of the SP from *Bifidobacterium adolescentis* using cross-linking by glutaraldehyde [11] instead of radical polymerization in pure organic solvents. Indeed, the use of an aqueous environment yields a considerably simpler imprinting procedure resulting in an imprinted cross-linked enzyme aggregate (iCLEA) exhibiting altered acceptor specificity as well as excellent stability at 60 °C.

## 2. Results and Discussion

### 2.1. Production of the iCLEA

**Figure 1:**
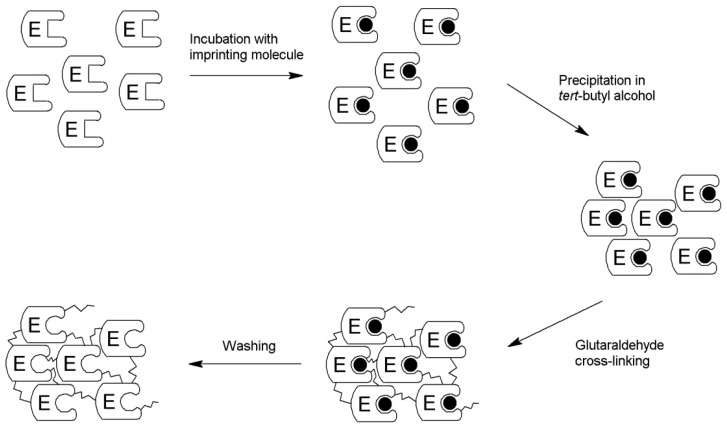
General scheme for the production of imprinted cross-linked enzyme aggregates (iCLEAs).

For the preparation of the imprinted cross-linked enzyme aggregate (iCLEA), the thermostable variant LNFI of the SP from *B. adolescentis* has been used [12]. The enzyme was recombinantly expressed in *E. coli* and partially purified by means of a heat treatment, which has been shown to remove the contaminating phosphatase activity [10]. The next steps in the preparation of the iCLEA then consist of imprinting, followed by precipitation (Figure 1).

To that end, the precipitation of SP in the presence of a number of possible imprinting molecules was first evaluated (Table 1). Previous research has identified *tert*-butyl alcohol as the precipitant of choice [10]. However, the presence of phosphate or α-G1P (100–500 mM) resulted in the appearance of a second phase upon addition of *tert*-butyl alcohol, excluding these molecules from use as imprinters. Also, addition of sucrose in a concentration higher than 200 mM was found to inhibit the precipitation of SP, whereas glycerol and αGG did not affect precipitation. Clearly, these results indicate the importance of evaluating precipitation thoroughly, prior to the production of an iCLEA.

Finally, the aggregated enzyme molecules were chemically cross-linked with glutaraldehyde to obtain the corresponding immobilizates. A typical immobilization yield of 30% has been reported for SP [10], and this value is also obtained in the presence of glycerol or αGG. However, the addition of sucrose during precipitation was found to increase the immobilization yield by roughly 10%. This result is in accordance with work published by Wang and collaborators, who reported a sugar-assisted precipitation strategy to increase the activity of a Penicillin G Acylase CLEA [22].

**Figure 2:**
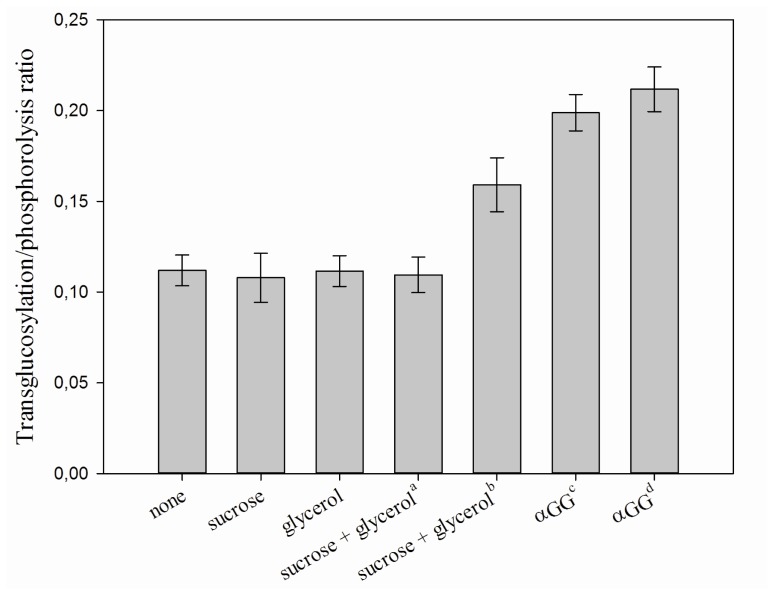
Transglucosylation/phosphorolysis ratio for different CLEA formulations. ^a^ Cross-linking was initiated upon addition of the imprinting molecules; ^b^ Cross-linking was initiated after 30 min incubation; ^c^ 200 mM α-glucosyl glycerol (αGG) was used; ^d^ 1 M αGG was used.

The specificity of the obtained formulations was then examined and compared with that of a non-imprinted CLEA. More specifically, their activity towards phosphate (phosphorolysis) and glycerol (transglucosylation) as acceptor was determined. Imprinting with sucrose, glycerol or a combination of both was found to have no influence on the specificity of the enzyme preparation (Figure 2). However, initiating aggregation and cross-linking after 30 min incubation with sucrose and glycerol did significantly increase the transglucosylation activity. This must mean that the presence of glycosylated product is required to modify the enzyme’s acceptor specificity. Indeed, imprinting with 200 mM αGG almost doubled the specific activity on glycerol, from 2.22 U/mg for the non-imprinted CLEA to 4.12 U/mg for the iCLEA. Increasing the αGG concentration to 1 M during imprinting. However, it did not significantly improve the acceptor specificity much further.

### 2.2. Characterization of the iCLEA

When SP is used for transglucosylation reactions, both primary and secondary hydrolysis can decrease the atom economy [4,23]. Although αGG itself is a very poor substrate, hydrolysis of sucrose can be quite significant and even lead to the formation of inhibiting products [5,24]. Therefore, the hydrolytic activity of the CLEA and iCLEA were examined in the presence of sucrose and glycerol as substrates. Hydrolysis was found to be identical for both biocatalysts, meaning that the ratio with transglucosylation is only half as high for the iCLEA than for the CLEA.

**Figure 3:**
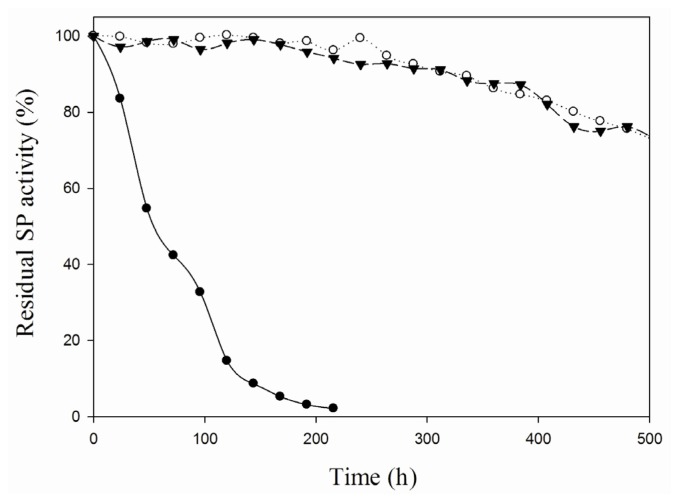
Stability of different SP formulations at 60 °C. The residual activity of soluble SP (●), CLEAs (○) and iCLEAs (▼) was measured every 24 h.

**Figure 4:**
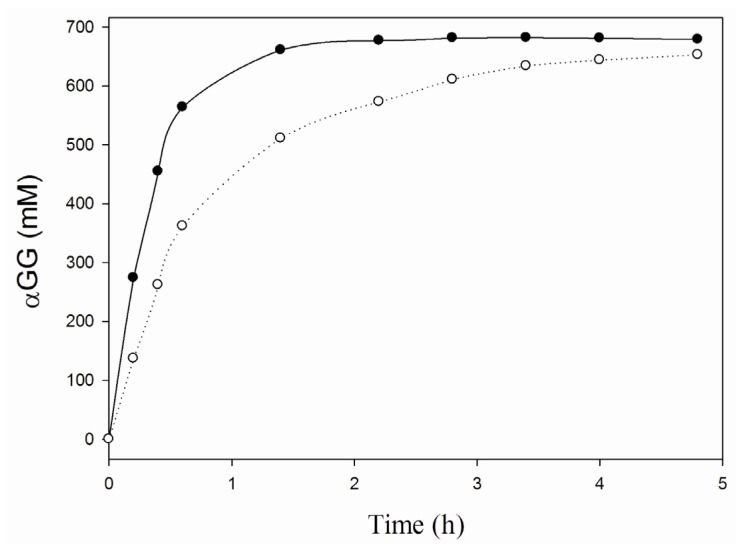
Synthesis of αGG with immobilized SP at 60 °C. The conversions were performed with 5 mg/mL CLEAs (○) or iCLEAs (●) in a 50 mM MOPS buffer pH 7 containing 800 mM sucrose and 2 M glycerol.

To further asses its applicability in carbohydrate conversions, the thermostability of the iCLEA preparation was evaluated by incubation at 60 °C and measuring the residual activity at several points in time (Figure 3). The iCLEA was found to exhibit a comparable thermostability to the non-imprinted CLEA, and thus to be drastically more stable than the soluble enzyme. Indeed, an impressive 75% of the initial activity was still present after three weeks incubation at 60 °C. Next, the efficiency of the newly formed iCLEA was demonstrated by comparing its performance with that of the reference CLEA in a batch production of αGG at 60 °C (Figure 4). This clearly showed that the maximal transfer yield of 84% [5] could be reached twice as fast with the iCLEA than with its non-imprinted counterpart. Furthermore, the newly formed iCLEA also proved its excellent operational stability by losing no activity whatsoever during five consecutive batch conversions (data not shown).

## 3. Experimental Section

### 3.1. Materials

Glucosyl glycerol (αGG) was produced as previously described [5]. *Tert*-butyl alcohol, glutaraldehyde, and sodium borohydride were purchased from Aldrich-Chemie (Bornem, Belgium), Fisher (Erembodegem, Belgium) and Acros (Geel, Belgium), respectively. All other reagents were analytical grade and purchased from Sigma-Aldrich.

### 3.2. Enzyme Production and Purification

*E. coli* XL10-Gold cells were used for transformation with the constitutive expression plasmid pCXshP34_BaSP_LNFI [12,25]. The resulting strain was routinely grown at 37 °C on 50 mL LB medium (10 g tryptone/L, 5 g yeast extract/L, 5 g NaCl/L; pH 7.0) supplemented with 100 mg/L ampicillin. After overnight growth, the culture was inoculated into 1 L of double LB medium (20 g tryptone/L, 10 g yeast extract/L, 5 g NaCl/L; pH 7.0) supplemented with 30 g/L glucose in a 2 L Biostat M reactor (B. Braun Biotech Inc., Allentown, PA, USA). Fermentation was done at 37 °C and 550 rpm with aeration at 1.2 vvm. When necessary, anti-foam was added manually to prevent foaming. After approximately 8 h of growth (OD_600_ was 22), the cells were harvested by centrifugation (7000 rpm, 4 °C, 20 min). The obtained pellets were frozen at −20 °C until further use.

Cell extracts were prepared by treatment of frozen pellets with 50 mM MOPS pH 7 supplemented with 100 mg/mL lysozyme for 30 min at room temperature, followed by 2 × 2 min sonification (Branson 250 Sonifier, level 3, 50% duty cycle). Subsequently, cell debris was removed by centrifugation (12,000 rpm, 4 °C, 30 min). Finally, the crude enzyme preparation was heat-purified by incubation at 60 °C for 60 min as described previously [10].

### 3.3. Preparation of the iCLEA

The heat-purified SP enzyme was diluted to a final protein concentration of 2 mg/mL and allowed to react with the imprinting molecules for 30 min at 37 °C. Next, 6 mL of *tert*-butanol was added to 4 mL of the imprinted SP solution under agitation. After 30 min, 1.36 mg glutaraldehyde was gently added to cross-link the enzyme aggregate, and the mixture was kept under stirring for 60 min. Reduction of the formed imine bonds was achieved by adding 10 mL of a solution containing 1 mg/mL sodium borohydride in 0.1 M sodium bicarbonate buffer at pH 10. After 15 min, another 10 mL was added and allowed to react for 15 min. Finally, the iCLEA was harvested by centrifugation (12,000 rpm, 4 °C, 30 min) and washed five times with 50 mM MOPS buffer at pH 7. All steps were performed in a thermoshaker (Eppendorf) at 750 rpm and 4 °C. The immobilization yield is defined as the ratio of the activity detected in the iCLEA preparation to that present in the original enzyme solution.

### 3.4. Activity Assays

Phosphorolysis of sucrose was measured discontinuously with an enzymatic assay, in which the production of αG1P is coupled to the reduction of NAD^+^ in the presence of phosphoglucomutase and glucose-6-phosphate dehydrogenase [26]. The reactions were analyzed by inactivation of samples (5 min at 95 °C) at regular intervals, followed by measurement of the absorbance at 340 nm in a microplate reader 680XR (Bio-Rad; Nazareth, Belgium). One unit of phosphorolysis activity corresponds to the release of 1 μmol fructose from 100 mM sucrose in 100 mM phosphate buffer at pH 7 and 37 °C.

The transglucosylation activity of SP towards glycerol was determined indirectly by measuring the increase in reducing sugars. More specifically, the release of fructose (and glucose) from sucrose as donor substrate was determined discontinuously with the bicinchonic acid (BCA) method [27]. In parallel, the release of glucose through hydrolysis was determined discontinuously with the glucose oxidase/peroxidase assay [28]. When glucose was detected, its concentration was subtracted twice from the concentration of reducing sugars measured with the BCA method, to calculate the net transglucosylation activity. One unit of transglucosylation activity corresponds to the production of 1 μmol αGG from 800 mM sucrose and 2 M glycerol in 50 mM MOPS buffer at pH 7 and 60 °C. Alternatively, the concentration of αGG was determined directly by HPLC analysis using an aminex HPX-87C column (Bio-Rad) with milliQ water as the mobile phase at a constant flow rate of 0.6 mL/min and 85 °C. The hydrolytic activity was quantified using 50 mM donor and 65 mM acceptor, which is identical to the conditions used to assess the transglucosylation potential of SP [8]. Protein concentrations were measured according to the Lowry method [29], using bovine serum albumin as standard. Unless otherwise stated, all experiments were performed in triplicate and had a CV of less than 10%.

### 3.5. Stability Assays

The thermostability of the iCLEA was determined by incubation in 50 mM MOPS buffer pH 7 in a water bath at 60 °C. Every 24 h, samples were inactivated and the residual activity was analyzed using the BCA method. In addition, the operational stability was evaluated by using the iCLEA for consecutive batch conversions of 800 mM sucrose and 2 M glycerol at 60 °C. After each reaction, the iCLEA were recuperated by centrifugation (12,000 rpm, 4 °C, 30 min) and washed three times with 50 mM MOPS buffer at pH 7.

## 4. Conclusions

Although molecular imprinting of a cross-linked enzyme aggregate (iCLEA) has been described for improving the activity of the hydroxynitrile lyase from *Linum usitatissimum* [30], this is the first time it has been used to alter the transglycosylation specificity of a biocatalyst. As proof of concept, sucrose phosphorylase was imprinted with α-glucosyl glycerol and subsequently cross-linking with glutaraldehyde. As a result, the enzyme’s specificity towards towards glycerol as acceptor was increased by two-fold. Furthermore, the obtained biocatalyst was extremely stable, and could be used for an industrially relevant production process at 60 °C. This convenient procedure thus allows us to simultaneously improve stability and specificity, and could find widespread application in the field of enzyme engineering.

## Figures and Tables

**Table 1:** Precipitation of sucrose phosphorylase (SP) in the presence of different imprinting molecules. Precipitation was achieved by adding 6 mL *tert*-butyl alcohol to 4 mL enzyme solution under agitation at 4 °C.

Imprinting molecule	[C] a(M)	Precipitation (%)	SP_residual_ b (%)
None	/	97.8	1.2
Sucrose	0.2	96.4	1.6
Sucrose	0.5	52.1	38.5
Sucrose	1	40.6	56.8
Glycerol	0.5	98.2	1.1
Glycerol	1	96.6	1.3
Glycerol	2	97.6	1.4
sucrose + glycerol	0.5 + 2	68.9	25.6
sucrose + glycerol	0.2 + 2	96.6	1.5
αGG	0.2	97.3	1.4
αGG	1	96.4	1.2

a of the imprinting molecules;

b after precipitation.
